# Encapsulation for Cancer Therapy

**DOI:** 10.3390/molecules25071605

**Published:** 2020-03-31

**Authors:** Xavier Montané, Anna Bajek, Krzysztof Roszkowski, Josep M. Montornés, Marta Giamberini, Szymon Roszkowski, Oliwia Kowalczyk, Ricard Garcia-Valls, Bartosz Tylkowski

**Affiliations:** 1 Department of Chemical Engineering, Universitat Rovira i Virgili, Av. Països Catalans 26, Campus Sescelades, 43007 Tarragona, Spain; marta.giamberini@urv.cat (M.G.); ricard.garcia@urv.cat (R.G.-V.); 2 Department of Tissue Engineering Chair of Urology, Ludwik Rydygier Collegium Medicum in Bydgoszcz Nicolaus Copernicus University in Torun, Karlowicza St. 24, 85-092 Bydgoszcz, Poland; a_bajek@wp.pl; 3 Department of Oncology, Nicolaus Copernicus University, Romanowskiej St. 2, 85-796 Bydgoszcz, Poland; roszkowskik@cm.umk.pl; 4 Eurecat, Centre Tecnològic de Catalunya. Chemical Technologies Unit, Marcel·lí Domingo s/n, 43007 Tarragona, Spain; josep.montornes@eurecat.org; 5 Faculty of Agronomy and Bioengineering, Poznan University of Life Sciences, Szydlowska St. 50, 60-656 Poznan, Poland; roszkowski.sz11@gmail.com; 6 Research and Education Unit for Communication in Healthcare Department of Cardiac Surgery, Ludwik Rydygier Collegium Medicum in Bydgoszcz Nicolaus Copernicus University in Torun, M. Curie Sklodowskiej St. 9, 85-094 Bydgoszcz, Poland; oliwiakowalczyk111@gmail.com

**Keywords:** cancer, nanomedicine, nanotechnology, nanocapsules (NCs), nanoparticles, anticancer therapy, bioimaging, drug delivery system, biocompatibility, biodegradability

## Abstract

The current rapid advancement of numerous nanotechnology tools is being employed in treatment of many terminal diseases such as cancer. Nanocapsules (NCs) containing an anti-cancer drug offer a very promising alternative to conventional treatments, mostly due to their targeted delivery and precise action, and thereby they can be used in distinct applications: as biosensors or in medical imaging, allowing for cancer detection as well as agents/carriers in targeted drug delivery. The possibility of using different systems—inorganic nanoparticles, dendrimers, proteins, polymeric micelles, liposomes, carbon nanotubes (CNTs), quantum dots (QDs), biopolymeric nanoparticles and their combinations—offers multiple benefits to early cancer detection as well as controlled drug delivery to specific locations. This review focused on the key and recent progress in the encapsulation of anticancer drugs that include methods of preparation, drug loading and drug release mechanism on the presented nanosystems. Furthermore, the future directions in applications of various nanoparticles are highlighted.

## 1. Introduction

Cancer is a group of diseases that entail an abnormal growth of malignant cells with the potential to invade or extent to other parts of the body [1,2]. Causes of cancer include smoking, obesity, intake of processed meat, radiation, family history, stress, environmental factors among other factors and humans can be affected by over 100 types of the cancer [3,4]. Each year, more than 10 million new cases of cancer are detected and the World Health Organization estimates that the cancer-related deaths are projected to increase to around 13.1 million by the year 2030 [5]. It is evident that cancer is one of the world’s most devastating diseases.

However, the mortality rate has decreased in the recent years owing to a better understanding of tumor biology that led to a significant progress in the prevention, detection, and treatment of cancer, yet effective therapy still proves challenging given the many variables such as type of cancer, detection stage or inadequate strategies in addressing aggressive metastasis as well as absence of/need for clinical procedures for tackling multidrug-resistant cancer [6]. The latest research is greatly focused on minimizing or improving effective therapies for cancer diseases which has been greatly recognized in the recent publication of seven articles in *Nature*. Thus, the results obtained by the international collaboration Pan-Cancer Analysis of Whole Genomes (PCAWG) will allow the identification by the international research community of common patterns of mutation in more than 2600 cancer whole genomes from the International Cancer Genome Consortium [7].

**Figure 1:**
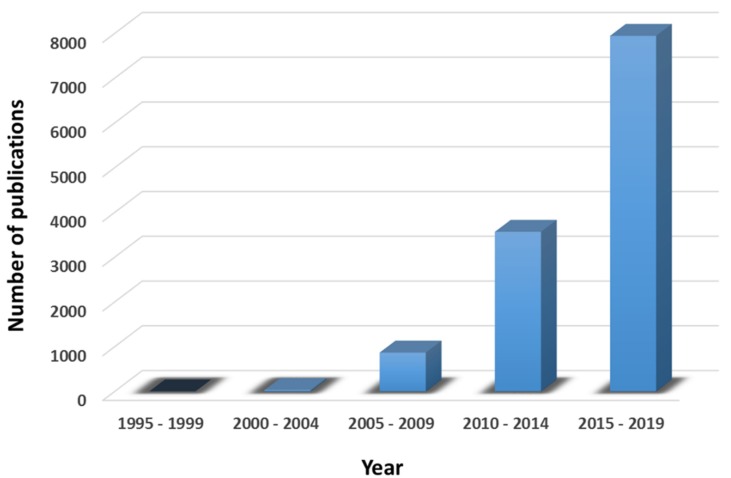
Number of peer-reviewed publications in the field of nanomedicine in Web of Science divided in periods of 5 years.

The exponential increase of the research and publications related to nanomedicine in the last decades is linked to the improvements observed in the field of medicine related to cancer therapy (Figure 1) [8,9,10]. The importance of research in cancer diseases has been also acknowledged by *Nature* editorial group that only recently published *Nature Cancer* journal focusing on exploration of leading edge solutions to cancer.

Nanomedicine is the medical application of nanotechnology [11,12]. Its use offers a variety of advantageous research tools and clinically useful devices due to the sizes of nanomaterials range from about one nanometer up to several hundred nanometers, comparable to many biological macromolecules such as enzymes, vitamins, hormones, antibodies and DNA plasmids. Some of these devices include NCs, biological devices and nanoelectronic biosensors. The similarity in size between these devices and biological molecules and structures makes them useful/favorable for both in vivo and in vitro biomedical research applications [13,14].

## 2. Benefits of the Encapsulation of Therapeutic Agents in Nanocapsules 

The encapsulation of therapeutic agents or drugs in different NCs offers a very promising system in advancement of nanomedicine. In general, nanotechnology is used in improving drug delivery efficacy as well as in addressing some of the limitations of drug delivery in cancer. The heterogeneous nature of cancers limit their treatment to chemotherapy, radiotherapy, immunotherapy, and surgery. Nonetheless, these approaches are sometimes restricted because they can be dangerous for healthy cells, they can damage the immune system or even they can increase the risk for the development of other types of cancers. Accordingly, one of the main goals of therapy research is aimed at finding effective therapies that can replace current therapies through the efficacy improvement and the reduction of possible side effects [15].

Besides, modern encapsulation methods exhibit other advantages over the conventional medical methodologies [16,17]:

- NCs have the ability to target and enter into selective tissue at molecular level.
- NCs provide large surface area.
- NCs provide high absorption rate.
- Increased cellular uptake and drug localization.
- Accurate and targeted drug delivery to cancerous cell without interactions with healthy cells.
- Lower dosage required due to the encapsulation of drugs or small molecules.
- Improved uptake of poorly soluble drugs.
- Decrease in medicinal toxicity.
- Greater precision in delivering drugs to tiny areas within the body.
- Decrease in drug resistance.
- Nanoencapsulation of the drugs minimizes or suppresses the resistance arising from the physiological barriers in the body.

## 3. Nanobased Drug Delivery Systems

**Figure 2:**
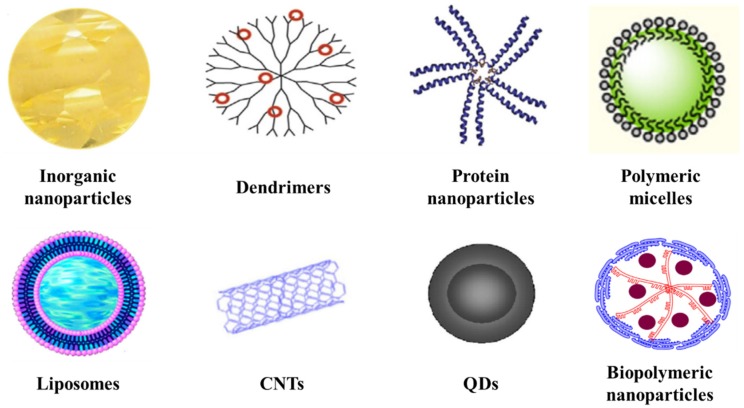
Examples of different nanoparticle drug delivery systems: inorganic nanoparticles, dendrimers, protein nanoparticles, polymeric micelles, liposomes, CNTs, QDs and biopolymeric nanoparticles.

In the past few decades, nanostructures with various shapes and sizes have been created and applied to encapsulate many drugs: inorganic nanoparticles, dendrimers, protein nanoparticles, polymeric micelles, liposomes, CNTs, QDs and biopolymeric nanoparticles (Figure 2). Table 1 summarizes the advantages and drawbacks of these nanoparticles. All these nanostructures, their properties and some examples of their applications related to cancer diseases are illustrated and discussed below [18]. 

There are different factors that influence the choice of the nanobased materials used for cancer diagnosis and drug delivery in cancer therapy:

- The size of the nanomaterial.
- The biocompatibility and biodegradability of the nanosystem.
- The desired drug release profile.
- The toxicity and antigenicity of the encapsulated drug.
- The properties of the entrapped medicine into the nanomaterial (for example drug stability or drug solubility in water or other solvents).

The current solution to the limitations arising from using each of these nanostructures alone stands in employing a combination of different nanostructures, resulting in the generation of hybrid nanoparticles [19,20].

### 3.1. Inorganic Nanoparticles

**Figure 3:**
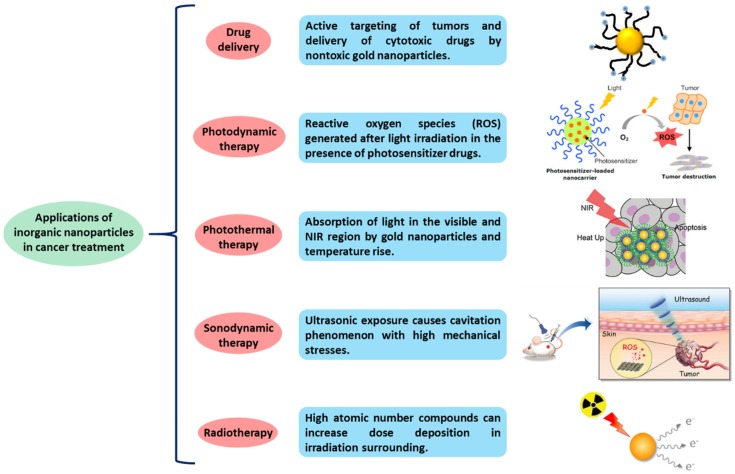
Applications of inorganic nanoparticles in cancer treatment.

Inorganic nanoparticles include gold [21], silver [22], iron oxide [23] and silica nanoparticles [24]. In recent years, the interest of using inorganic nanoparticles has been growing in different medical applications, such as bioimaging, biosensors, target/sustained drug delivery and photoablation therapy due to their particular properties like surface plasmon resonance (SPR). The SPR phenomenon enables inorganic particles to convert light into heat and scatter the produced heat to destroy the cancer cells by irradiating previously the nanoparticles [25]. Furthermore, the surface functionalization of inorganic nanoparticles provides them with a good biocompatibility and versatility, making these systems more promising for their use in biomedical applications [26]. Some of the applications in which inorganic nanoparticles are used for cancer treatment are summarized in Figure 3. 

Among all inorganic nanoparticles, gold nanoparticles show exceptional optical, electronic, sensing and biochemical properties that make them potentially effective in medical imaging, drug delivery, and tumor therapy in early cancer detection. Other properties like good stability, facile synthesis, high biocompatibility combined with low toxicity and the reactivity of their surface makes gold nanoparticles ideal candidates for its use in medical activities. Anticancer drugs can be conjugated to the surface of gold nanoparticles via physical absorption or via ionic or covalent bonding. Related to the delivery of anticancer drugs from gold nanoparticles, different investigations showed that it can be controlled through biological stimuli or via an external activation [27].

**Figure 4:**
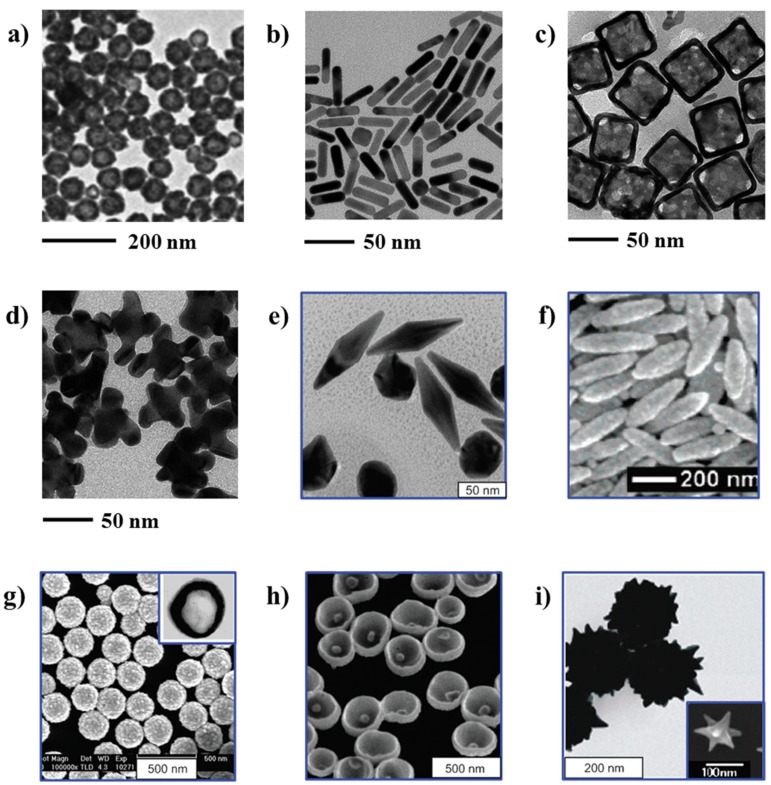
TEM images of different types of gold nanoparticles: (**a**) gold nanospheres; reprinted with permission from [30]; copyright (2005) American Chemical Society; (**b**) gold nanorods; reprinted with permission from [31]; copyright (2013) American Chemical Society; (**c**) gold nanocages; reprinted with permission from [31]; (**d**) gold nanohexapods; reprinted with permission from [31]; (**e**) gold bipyramids; reprinted with permission from [32]; copyright (2005) American Chemical Society; (**f**) “nanorice” (gold-coated Fe_2_O_3_ nanorods); reprinted with permission from [33]; copyright (2006) American Chemical Society; (**g**) SiO_2_/Au nanoshells (the inset shows a hollow nanoshell); reprinted with permission from [29]; copyright (2010) Elsevier; (**h**) nanobowls with bottom cores; reprinted with permission from [34]; copyright (2009) American Chemical Society; (**i**) spiky SiO_2_/Au nanoshells (reprinted with permission from [35]; copyright (2010) American Chemical Society; the inset shows a gold nanostar); reprinted with permission from [36]; copyright (2006) American Chemical Society.

Traditionally, the synthesis of gold with quasispherical shapes (nanospheres) was reported in literature for microscopic studies related to cancer treatment [28]. Nonetheless, in recent years the synthesis of gold nanoparticles has been tuned in order to obtain particles with controlled sizes, shapes and structures, which determine the unique optical and electrical properties of the resulting nanoparticles [29]. The TEM pictures of several gold nanostructures, potentially encouraging in cancer theranostics (contrast-enhanced diagnosis and photothermal cancer therapy) are shown in Figure 4.

Another kind of inorganic nanoparticles that are widely used for the encapsulation of anticancer medicines are iron oxide nanoparticles. In one of their studies, Chen et al. reported the preparation of Fe_3_O_4_-doxorubicin (Fe_3_O_4_-DOX) inorganic nanoparticles that were coated with a layer of silica and polyethylene glycol (PEG), respectively. The resulting drug delivery system could work as a magnetic carrier due to the amount of doxorubicin (DOX) released during transportation prior to release is slower compared to DOX-conjugated Fe_3_O_4_ nanoparticles alone in targeting tumor and exhibits a high sensitivity to external magnetic fields. Moreover, it can be easily post-functionalized with targeting ligands [37]. 

On the other hand, silica nanoparticles are encouraging candidates for tumor targeted drug delivery due to their advantages versus other inorganic nanoparticles: the payload and release profile of drugs in gold and iron oxide nanoparticles is deficient due to the solid nature of these nanoparticles and the improved biodegradability of silica nanoparticles [38]. 

Within the silica nanoparticles, mesoporous silica nanoparticles (MSNs) have attracted much attention due to their unique physicochemical properties: adjustable particle size (from 10 nm to micron range) and pore size (from 2 to 50 nm), high specific pore volume and surface area combined with their low cytotoxicity. The possibility to adjust their pore size allows drugs of different shapes and sizes to be loaded. Furthermore, the surface of MSNs is rich in silanol groups, which can be modified with a wide range of functional groups or molecules: polymers, metals, metal oxides, targeting ligands, among others, defining the final application of MSNs (tumor targeting, stimuli responsive release, bio-imaging, etc.) [39,40].

Geng et al. described the synthesis of hollow MSNs with controlled shape. By employing Fe_2_O_3_ as hard template, the authors obtained different hollow MSNs that exhibit a controlled release behavior of DOX by the application of an acidic pH. In the same study, the modification of the surface of MSNs with PEG and Folic acid supply an efficient and tumor-cell-selective drug-delivery system towards HeLa and A549 cells [41]. 

Moreover, a large number of studies reported the use of silica as a shell to cover different kind of nanoparticles, as can be seen in the study described previously by Chen et al. [37]. Additionally, researchers started to report the application of silver nanoparticles in drug delivery for cancer treatment in the recent years. Some of the properties that make silver nanoparticles good candidates for potential applications in cancer are: relative lower toxicity and biological activities such as antimicrobial, inflammatory, and intrinsic therapeutic properties [42,43,44].

### 3.2. Dendrimers

Dendrimers are repetitively highly branched polymers with a defined diameter in the order of nanometers with a spherical 3D morphology. The term dendrimer comes from the Greek words “Dendron”, which means a tree, and “Meros”, which means a part. Since the discovery of dendrimers in 1978 by Buhleier et al. [45], their chemistry has rapidly evolved very fast in different areas (biomedical applications like drug or gene delivery as well as sensors). The structure of the dendrimers is divided in three different parts:

- The internal core.
- Branches: The interior layers or also called generations composed of repeating units.
- Surface moieties: Outer part, which involves the peripheral end groups of the most external generation.

Three different approaches can be used for the synthesis of dendrimers:

- Divergent approach: Dendrimer grows from a multifunctional core molecule towards periphery.
- Convergent approach: The dendrimer formation starts from the peripheral end and it progresses towards the core.
- Double stage convergent approach: The building blocks are synthesized by divergent method followed by convergent dendrimer assembly.

**Figure 5:**
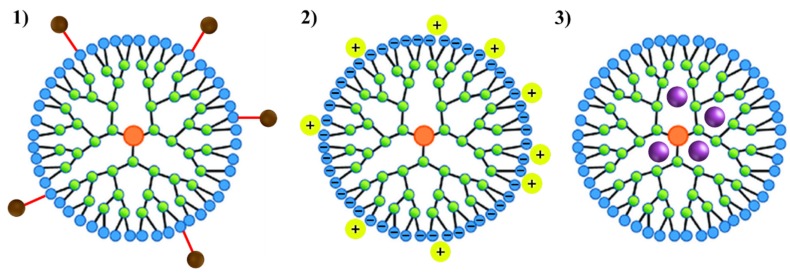
Schematic representation of the 3 mechanisms used to load drugs in dendrimers: (**1**) through a covalent interaction between the drug to the periphery of the dendrimer; (**2**) by coordination of the drug to the outer functional groups of the dendrimer via ionic interactions; (**3**) by simple encapsulation of the drugs into the dendrimer cavities.

The three synthetic methods mentioned above permit the control of the critical parameters such as size, shape, surface/interior chemistry, flexibility and topology, allowing the synthesis of well-defined structures with good properties (higher chemical and biological stability, efficacy, purity and long shelf life) [46]. This combination of properties make dendrimers interesting materials for drug delivery applications. The loading of dendrimers with medicines can be performed by distinct mechanisms: (1) through a covalent interaction between the drug to the periphery of the dendrimer, (2) by coordination of the medicine to the outer functional groups of the dendrimer via ionic interactions or (3) by simple encapsulation of the drugs into the dendrimer cavities [47]. These three possibilities are depicted in Figure 5.

**Figure 6:**
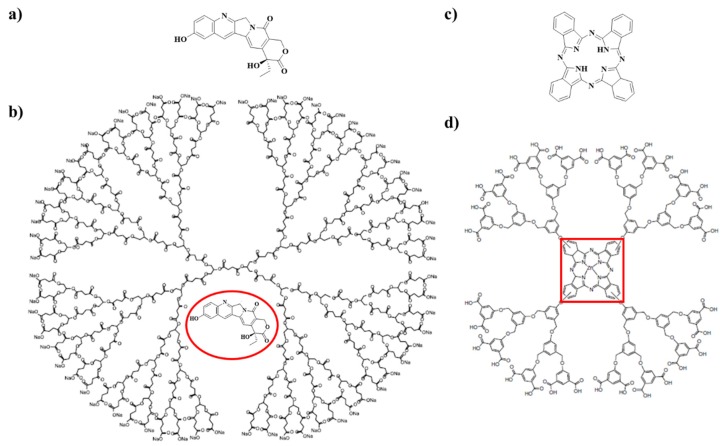
(**a**) Chemical structure of 10-hydroxycamptothecin (10HCPT), (**b**) Chemical structure of a poly(glycerol-succinic acid) (PGLSA)-COONa dendrimer with encapsulated 10HCPT, (**c**) phthalocyanin, a photosensitizer for non-invasive cancer treatment and (**d**) zinc phthalocyanin covalently conjugated to a dendrimer structure. Not drawn to scale.

The chemical structure of two anticancer drugs and the dendrimers used for their encapsulation are depicted in Figure 6. Morgan et al. reported the encapsulation of camptothecins, which are used to treat different kinds of cancer (colon, ovarian and lung cancer), in dendrimers [48]. In their research, the authors synthesize a well-defined, single molecular weight dendrimers with a polyester based structure composed of succinic acid and glycerol: poly(glycerol-succinic acid)-COONa ((PGLSA)-COONa). Thus, the obtained biodegradable and biocompatible dendrimers increase aqueous solubility of the derived camptothecin drugs and improve both the uptake and retention of camptothecins within cancer cells (Figure 6a,b). Figure 6c shows the chemical structure of zinc phthalocyanine, which is used for tumor targeting and treatment [49,50,51]. The covalent conjugation of zinc phthalocyanine with a dendrimeric structure results in a controlled drug delivery system that could be photochemically activated [52]. 

The use of different types of dendrimers in medical applications are reported in literature: polyamidoamine (PAMAM), polypropylene imine (PPI) and poly-L-lysine (PLL) dendrimers are the most used, among others. However, the presence of amine groups in the surface of the dendrimeric structures usually limits their medical applications due to their toxicity. Therefore, the outer layer of dendrimers are functionalized with PEG or other biological macromolecules like antibodies or carbohydrates to reduce or suppress their toxicity [53].

**Figure 7:**
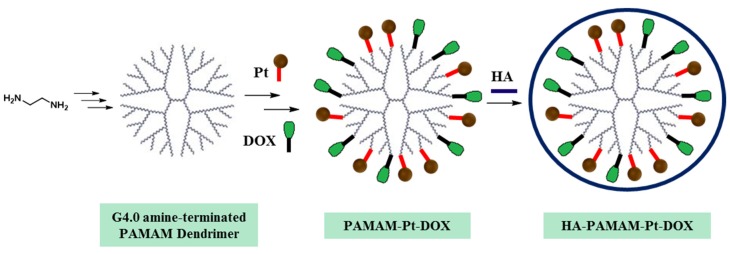
Scheme of the synthesis of PAMAM dendrimers with two drugs (Pt and DOX) covalently linked to the amine groups of the outer generation of the dendrimer. The addition of an external shell of HA, which is coordinated through electrostatic interactions with PAMAM, improve the biocompatibility of the dendrimers.

In a recent published example, Guo et al. reported the preparation of a new PAMAM dendrimer that can be used as controlled drug delivery system [54]. As depicted in Figure 7, two anticancer drugs were covalently bonded to the dendrimer, which is then coated with Hyaluronic acid (HA) to improve the stability and biocompatibility of the nanodrug system apart from acting as targeting group to tumor site. The two anticancer drugs used in this study are:

- Cisplatin (Pt): an anticancer drug used in chemotherapy to treat diverse types of cancer like testicular cancer, ovarian cancer, cervical cancer, breast cancer, bladder cancer, head and neck cancer, esophageal cancer, lung cancer and brain tumors.
- Doxorubicin (DOX): an anticancer drug from the anthracycline family. Applied in the treatment of distinct human tumors (bladder, stomach, ovaries, lung, and thyroid among others).

The application of an external stimulus (an acidic pH) has a remarkable effect in the release of Pt and DOX from the dendrimers. Furthermore, the resulting PAMAM-Pt-DOX nanoparticles coated with HA exhibited a higher efficiency to kill breast cancer cells and display a lower toxicity of DOX compared with PAMAM-Pt-DOX dendrimers.

### 3.3. Protein Nanoparticles

Proteins are considered natural biopolymers since they are extracted from biological sources such as plants (zein, gliadin and soy protein) or animals (gelatin, collagen, albumin, casein and silk protein). Protein nanoparticles have gained great interest in nanotechnology because of their excellent properties: low toxicity, biodegradability, versatility, great similarity with components of the extracellular matrix and solubility in water in the case of bovine and human serum albumin proteins [55]. 

Protein nanoparticles can be synthesized by different methods: coacervation/desolvation, emulsion/solvent extraction, complex coacervation and electrospraying. The chemical functionalization of the surface of protein nanoparticles through the addition of targeting ligands such as peptides, antibodies, vitamins, hormones or enzymes, that identify exact cells and tissues promotes and improve their targeting mechanism [56]. 

**Figure 8:**
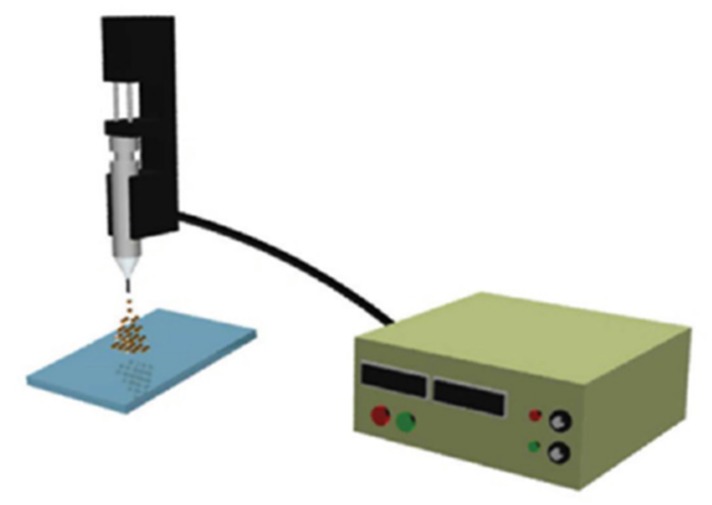
Scheme of the electrospray deposition system to synthesize gliadin-based nanoparticles. Reprinted with permission from [57]; copyright (2012) American Chemical Society.

Gulfman et al. reported the synthesis of cyclophosphamide-loaded nanoparticles from natural gliadin and gelatin by using an electrospray deposition system (Figure 8). The study proved that gliadin nanoparticles facilitate the delivery and controlled release of cyclophosphamide anticancer drug in a controlled manner to induce apoptosis in breast cancer cells compared with gliadin-gelatin composite nanoparticles, from which cyclophosphamide was released in a rapid manner [57].

**Figure 9:**
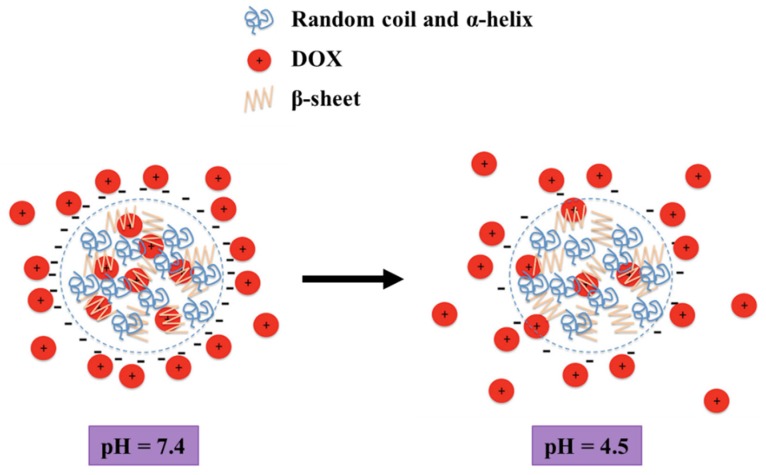
Schematic representation of silk nanoparticles and pH-dependent release of DOX. Diagram not drawn to scale. Reprinted with permission from [58]; copyright (2013) Wiley Online Library.

Despite silk has been traditionally associated to the preparation of materials for tissue engineering in medicine due to their excellent mechanical properties and biocompatibility, its potential for the construction of drug delivery complexes is growing a lot nowadays. In the last years, *Seib* et al. developed the preparation of silk nanoparticles loaded with DOX. By precipitation of silk in acetone, the formation of nanoparticles with a well-defined nanoscale structure were achieved. The use of the resulting silk nanoparticles for drug delivery displayed a pH-dependent release of DOX, which release takes place faster at acidic pH than at pH around 7 (Figure 9). Besides, DOX loaded silk nanoparticles showed reduced drug resistance mechanisms compared with DOX alone due to the higher biocompatibility of silk, which also demonstrate a lysosomal accumulation in breast cancer cells. These results, combined with the non-cytotoxic character of silk based nanoparticles, proved that these nanoparticles can be used as controlled drug delivery systems to heal cancer by the application of an external stimulus [58].

### 3.4. Polymeric Micelles

Polymeric micelles are nanosized colloidal particles with a core-shell structure, which are spontaneously formed by the self-assembly of amphiphilic macromolecules in a block-selective solvent into nanoaggregates above a certain concentration, the critical micelle concentration (CMC). Amphiphilic block copolymers assemble in water into various supramolecular nanostructures for drug delivery: cylindrical micelles, spherical micelles and vesicles [59]. Usually, PEG is used to stablish the shell of the micelle due to their low toxicity [60]. On the other hand, there are different hydrophobic polymers that can be used in the core of the micelle: poly (D,L-lactic acid) (PLA), poly(α-amino acid), pluronics, polycaprolactones (PCL) among others. 

**Figure 10:**
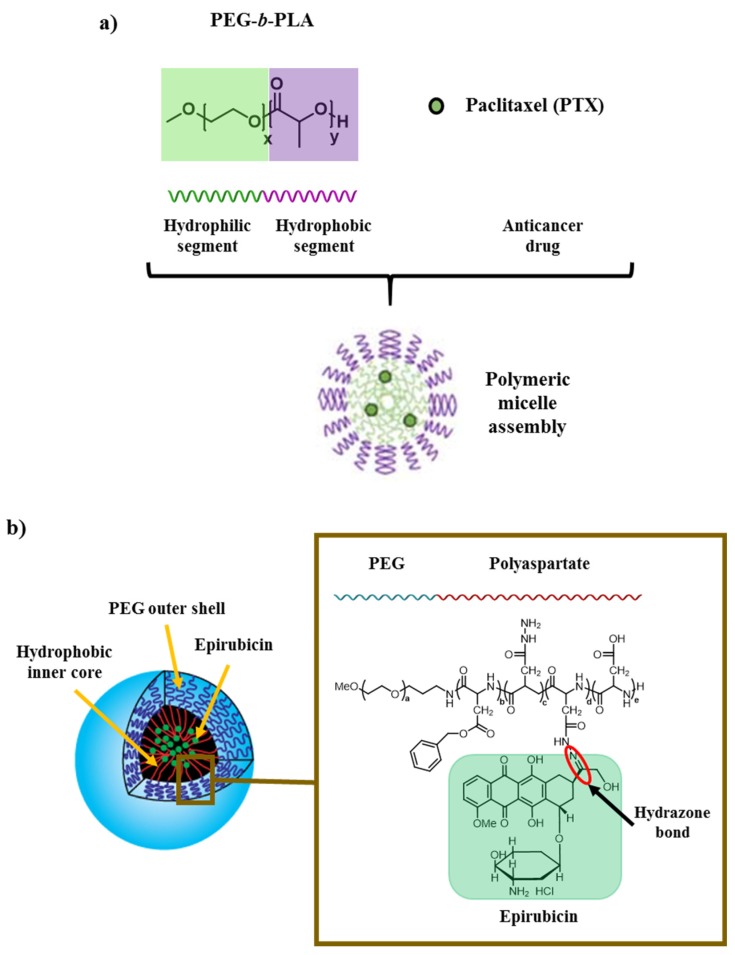
(**a**) Solubilization of PTX in a polymeric micelle made of amphiphilic block copolymers (Physical loading of anticancer drug into the micelles). Adapted with permission from [63]; copyright (2007) American Chemical Society. (**b**) Inner structure of micelles with encapsulated Epirubicin covalently linked to the polyaspartate chain of PEG-polyaspartate block copolymer by an acid-labile hydrazone bond. The amphiphilic block copolymers (PEG and polyaspartate) forms spontaneously micellar structures in an aqueous media.

Anticancer agents can be loaded physically or chemically in the core region of the polymeric micelles. The encapsulation in a polymeric micelle of paclitaxel (PTX), a drug used for the treatment of different kinds of cancer (ovarian cancer, breast cancer, lung cancer, cervical cancer, pancreatic cancer and Kaposi sarcoma), is illustrated in Figure 10a [61]. PEG-*b*-PLA micelles are widely used to increase the poor solubility of different drugs like PTX. In this case, micelles were loaded physically with the anticancer drug. 

Figure 10b depicts the loading of epirubicin, which is used for chemotherapy to treat various human tumors (breast, ovarian, gastric and lung cancer and lymphomas), into polymeric micelles. Epirubicin belongs to the family of the anthracyclines such as DOX and is covalently bonded through an acid-labile hydrazone bond into PEG-polyaspartate diblock copolymer in the resulting micelles. Nevertheless, epirubicin exhibit side effects among which the most prominent is cardiotoxicity. By the encapsulation of epirubicin, its cardiotoxicity is reduced without loss of the antitumor effect [62]. 

Because many of the anticancer drugs have hydrophobic character, the use of polymeric micelles in clinical applications for the treatment of cancer has increased considerable in the last few decades. Nowadays, several polymeric micelles formulations are being evaluated in clinical trials, among which one of them (Genexolt-polymeric micelle) has been FDA approved [64].

**Figure 11:**
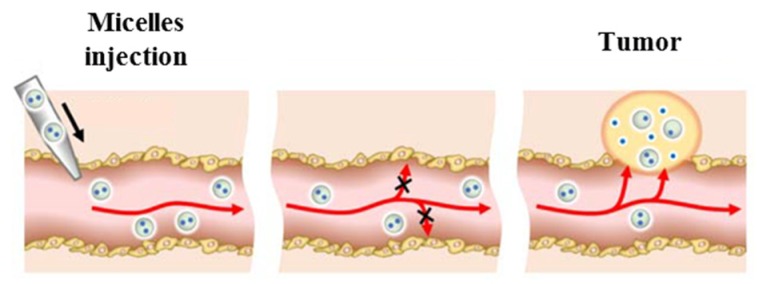
Cancer drug delivery via the injection of drug-loaded polymeric micelles. Adapted with permission from [61]; copyright (2016) Springer.

Since many of the encapsulated drugs in micelles used to treat cancers (like PTX) are usually administered by intravenous therapy, Figure 11 describes the path that the polymeric micelles loaded with anticancer drugs follows via intravenous injection. The administered micelles are stable against drug precipitation or direct drug absorption in non-carcinogenic tissues. Once the polymeric micelles accumulate at the targeted tissue, the drugs are released by the application of some stimulus: pH, temperature, magnetic field, light, ultrasound, enzymes, etc. Moreover, polymeric micelles replace the first toxic vehicles used in biomedical applications like Cremophor EL or ethanol due to their improved retention and stability of anticancer drug-loaded polymeric micelles upon injection [61].

### 3.5. Liposomes

Liposomes are one of the most studied systems for drug delivery in biomedical applications, which includes cancer diseases. Moreover, several examples are reported in which liposomes are used in pharmaceutical and cosmetics industry to transport different small molecules [65,66]. Liposomes, which were discovered by Alec Bangham in 1960, are spherical vesicles with a phospholipid bilayer [67,68]. Phospholipids are a major component of all cell membranes and consists of two hydrophobic fatty acid “tails” and a phosphate-based hydrophilic “head”. 

Different types of drugs have been encapsulated on liposomes especially to increase their efficacy and specificity. Depending on the drug that will be encapsulated and their possible application, different kind of lipids can be used in the synthesis of liposomes: cationic, anionic and neutral lipids. 

However, conventional liposomes have some limitations for their use in biomedical applications: (instability, insufficient drug loading, faster drug release and shorter circulation times in blood). To minimize or suppress these drawbacks, liposomes can be functionalized. One option involve the synthesis of PEGylated liposomes in order to generate a steric barrier [69]. Other options include the use of different ligands like antibodies, carbohydrates and peptides, which are linked to the surface of the liposome [70]. The liposomes with grafted ligands can actively target the cancer sites [71,72].

The free use of DOX is connected with the appearance of different toxicities: gastrointestinal myelosuppression, alopecia and mucositis. Kale et al. reported that the encapsulation of DOX in liposomes reduces its toxicity and also increases its retention time [73]. In another studies, the encapsulation of DOX in liposomes delayed tumor growth [74].

**Figure 12:**
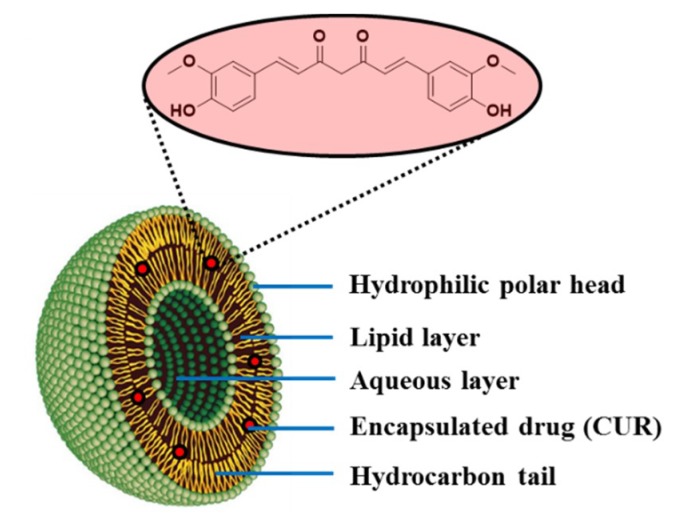
Schematic representation of the inner structure of a liposome drug delivery system with encapsulated CUR.

The encapsulation of curcumin (CUR) in a liposome is shown in Figure 12. CUR is a natural compound extracted from the plant turmeric, which is widely used to treat many types of cancers such as those of lung, cervices, prostate, breast, bone and liver [75]. However, the administration of free CUR have some limitations: poor aqueous solubility and low bioavailability. In the last decade, several authors reported the encapsulation of CUR in liposomes employing different methodologies to provide effective drug delivery systems in clinical practice to treat cancers [76]. The most common methods for the encapsulation of drugs in liposomes are the thin film method and the ethanol injection method. In 2017, Cheng et al. developed an organic solvent-free and easily scalable encapsulation technique that involves the pH-dependent solubility properties of CUR. By this method, CUR was deprotonated and dissolved under alkaline conditions before than been encapsulated into the liposomes after acidification. The CUR loaded liposomes prepared by the pH-driven combine the advantages showed by the liposomes prepared by the thin film and by the ethanol injection methods: (1) They are stable under storage and (2) They showed high bioaccessibility [77]. 

### 3.6. Carbon Nanotubes (CNTs)

CNTs are carbon cylinders with a diameter in the nanometer size made of graphene sheets. Depending on the number of graphene layers from which a single nanotube is composed, CNTs are classified as single-walled carbon nanotubes (SWNTs) or multi-walled carbon nanotubes (MWNTs). 

CNTs have been investigated as an excellent candidate for drug delivery carrier due to their quasi 1D nanostructure, their remarkable optical and electronic properties, exceptional surface area, which allows efficient loading of multiple molecules along the length of the nanotube sidewall, and cell membrane penetrability. Moreover, CNTs can be easily functionalized by different ways, which increases its compatibility with medicines and with human cells [78,79,80].

In the last decades, the usage of CNTs were investigated in different biomedical applications: drug delivery, cancer therapies, biosensing, bioimaging, and tissue engineering [80,81]. For instance, different CNT-based in vivo photothermal therapy of cancer in animal models has been reported in several investigations in the last decade because SWNTs are highly absorbing materials with strong optical absorption in near-infrared spectroscopy (NIR) and RAMAN spectroscopy [82,83,84]. Generally, anticancer drugs can be attached to CNTs by covalent or non-covalent interactions between the drugs to the functionalized CNTs.

Related to the covalently bonded anticancer drugs to CNTs, Liu et al. were among the first authors to describe CNTs-based in vivo drug delivery for cancer treatment in animal experiments. They reported that PTX was chemically conjugated to branched PEG chains on SWNTs via a cleavable ester bond. When they treated the 4T1 murine breast cancer in mice with different forms of PTX for some weeks, an improvement of the blood circulation time of the SWNT-PTX complex was extended in time when compared with free PTX. As a result, an increased drug accumulation of PTX in the tumor was achieved, which results in an enhancement of the therapeutic efficacy for the delay of tumor growth [85]. Other representative examples in which the authors covalently linked CNTs with antitumor agents were reported in literature during the last decades by Wu et al. and Bhirde et al., respectively [86,87].

**Figure 13:**
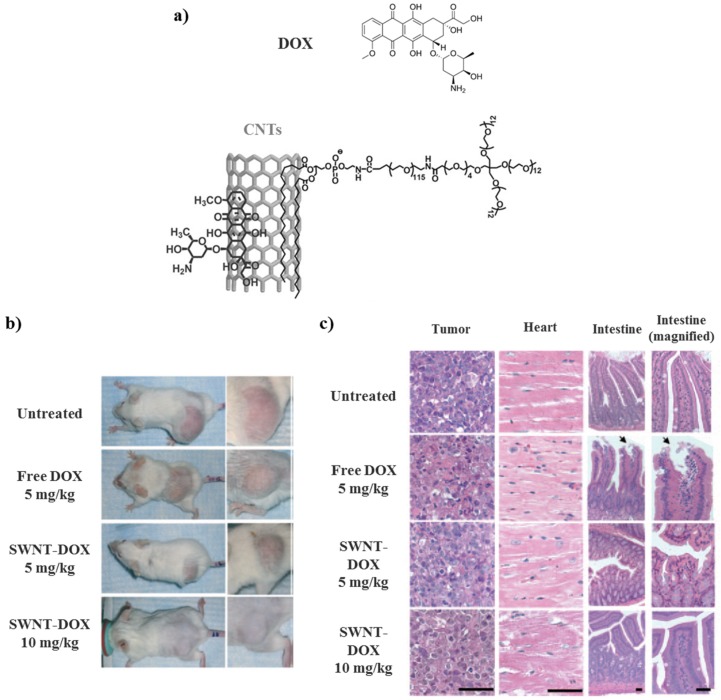
Schematic illustration of drug delivery systems comprising SWNTs. (**a**) A scheme showing non-covalent supramolecular π–π stacking of DOX with SWNTs. (**b**) Representative photos of mice from different groups were taken after treatments with DOX. (**c**) Gastrointestinal toxicity was observed in mice treated with free DOX but not in mice treated with SWNT-DOX. Histological sections of intestinal epithelium showed damage of the intestinal epithelium in the free DOX treated group. The arrows show the area of loss of columnar epithelial cells in tips of villi. (Scale bars: 100 micrometers). Reprinted with permission from [88]; copyright (2009) Wiley-VCH.

Conversely, Liu et al. described the build of a drug delivery system based on SWNTs in which DOX was non-covalently linked to CNTs. The resulting system could improve the therapeutic efficacy and reduce drug-related toxicities. They demonstrated that DOX could be loaded onto branched PEG functionalized SWNTs by using supramolecular π–π stacking (Figure 13a). The treatment of tumor with SWNT-DOX showed less in vivo toxicity when compared with free DOX. The reduced toxicity of SWNT-DOX might allow the administration of higher doses of DOX, which ameliorate the treatment efficacy (Figure 13b,c) [88]. 

### 3.7. Quantum Dots (QDs)

QDs are nanoscale semiconductor crystals composed of a core of groups II-VI or III-V elements and a shell with polymer coating, which protects the metal crystal core [89,90]. Although the QDs were discovered in the early 1980s by Ekimov and Efros [91], the interest in their use for different biomedical applications has grown considerably due to their advantages in front of other materials:

- Low toxicity compared to inorganic nanoparticles.
- Strong fluorescence intensity compared to organic fluorophores in biomedical imaging.
- Improved aqueous solubility upon surface modification.

These properties make QDs useful for imaging applications to allow cancer detection. For instance, Shi et al. developed multifunctional biocompatible graphene oxide QDs covered with luminescent magnetic nanoplatform for the recognition/diagnostic of a specific liver cancer tumor cells [92]. Moreover, more examples of their use as therapeutics started to develop in the last years [93,94].

**Figure 14:**
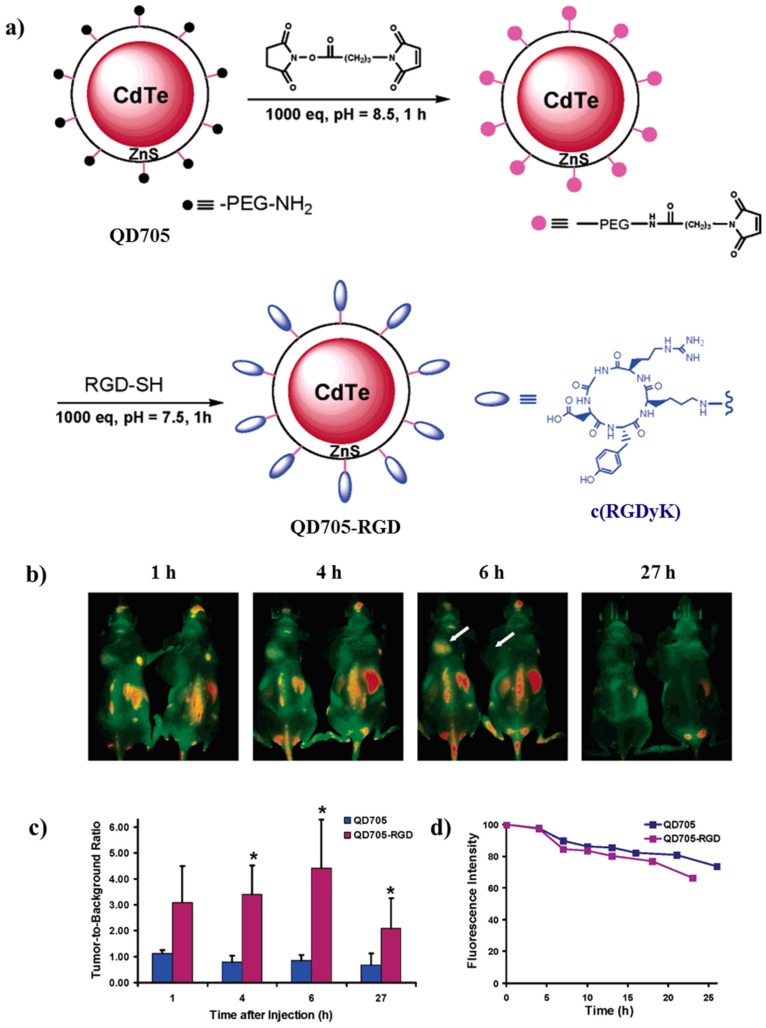
(**a**) Synthetic pathway for the preparation of QD705-RGD. (**b**) In vivo NIR fluorescence imaging of U87MG tumor-bearing mice (left shoulder, pointed by white arrows) injected with 200 pmol of QD705-RGD (left) and QD705 (right), respectively. All images were acquired under the same instrumental conditions. The mice autofluorescence is color coded green while the unmixed QD signal is color coded red. Prominent uptake in the liver, bone marrow, and lymph nodes was also visible. (**c**) Tumor-to-background ratios of mice injected with QD705 or QD705-RGD. The data were represented as mean (standard deviation (SD)). Using one-tailed paired Student’s t-test (*n*) 3),”*” denotes where *p* < 0.05 as compared to the mice injected with QD705. (**d**) Serum stability of QD705 and QD705-RGD in complete mouse serum over the course of 24 h. Reprinted with permission from [95]; copyright (2006) American Chemical Society.

One of the first works in which the authors demonstrated the use of QDs for tumor imaging was published by Cai et al. In this study, they build up a tripeptide-tagged (arginine-glycine-aspartic, RGD) CdTe/ZnS QDs that present particular targeting properties in tumor vessels in vivo (Figure 14a). In the in vivo studies, a well-known dose of QD705 or QD705-RGD were administered via tail vein injection to mice that contains the U87MG tumor. Figure 14b shows that after 20 min of the injection of QD705-RGD, a fluorescence signal was observed in the tumor. From that point, the tumor fluorescence intensity in mice injected with QD705-RGD increases as observed in Figure 14c until 6 h after the injection, in which the intensity signal reached its maximum. 

Differently, no significant fluorescence signal was observed in the tumor for the QD705 (Figure 14c). On the other hand, both QDs were incubated in complete mouse serum at 37 °C to prove the fluorescence intensity throughout the time (Figure 14d). Both systems showed a decrease in fluorescence intensity, which can be associated to the decrease in tumor contrast at late time points. Hence, the good fluorescence intensity in the NIR of QD705-RGD can be used in fast cancer diagnosis or surgery by the integration of targeted NIR optical imaging in cancer therapy [95].

**Figure 15:**
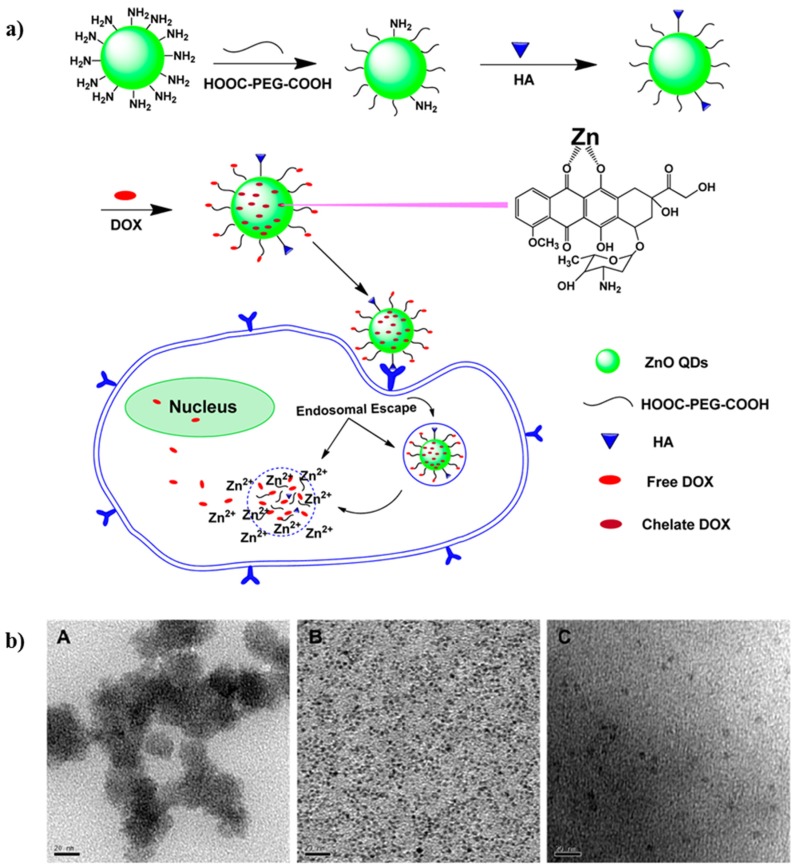
(**a**) Synthetic pathway and assay of working protocol of the Hyaluronic Acid (HA)-ZnO QDs-Dicarboxyl-Terminated PEG drug delivery system. (**b**) TEM images of: (**A**) ZnO QDs, (**B**) NH_2_-ZnO QDs, and (**C**) PEG-ZnO QDs. The scale bar represents 20 nm. Reprinted with permission from [99]; copyright (2016) American Chemical Society.

On the other hand, there are some examples in literature that reports the usage of QDs for the controlled delivery of anticancer drugs via external stimulus (light, heat, radio frequency or a magnetic field) [96,97,98]. As an example, Cai and coworkers demonstrated a controlled drug delivery system for cancer of ZnO QDs. Specifically, they prepared a pH-sensitive QDs system for the delivery of DOX as drug for cancer therapy. These QDs remained stable at physiological pH because they were coated with PEG. DOX was loaded to the resulting QDs by conjugation of the drug to PEG or by forming a complex with Zn^2+^ cations, which can be biodegraded completely in the acidic environment of tumors. Once the QDs were biodegraded, the released DOX can kill cancer cells. They proved that the combination of DOX with ZnO QDs improves the apoptosis of cancer cells (Figure 15) [99].

Despite the good results observed with QDs, their application in lung cancer imaging has still some limitations related with their wide use like cytotoxicity to lung cells and induction of oxidative stress [100].

### 3.8. Biopolymeric Nanoparticles

Nature has always been a source of inspiration for humans. In research, scientists try to extract guiding principles from nature in order to design and provide novel biomaterials for many years, which allow us to live in a better and more sustainable society. In fact, nature is plentiful of different materials that can be used in nanomedical devices [55]. During the last decades, there has been an increasing interest in the utilization of sustainable natural resources due to their potential for the synthesis of high-value products with low environmental impact. Natural biopolymers could be obtained from different resources:

- Higher plants: Starch, cellulose, guar gum, gum arabic.
- Animals: Chitin, chitosan, glycosaminoglycans, hyaluronic acid.
- Microorganisms: Dextran, gellan gum, xanthan gum, bacterial cellulose.
- Algae: Alginate, galactans, carrageenan.

From among all of them, chitosan, cellulose and alginate are the most widely used natural polymers in medicine due to their geometrical dimensions, high specific surface area, their mechanical and barrier properties, lack of toxicity, biocompatibility and biodegradability, etc. All these properties make natural polymers and excellent candidates for their utilization in nanomedicine [101,102].

Cellulose can be isolated from different natural resources: plants, wastes, animals (tunicates), bacteria and algae. Its abundance make that cellulose nanomaterials are considered an important candidate for the preparation of drug delivery systems that could be used for cancer treatment. As proof of this, the number of publications based on this subject have experienced a considerable increase during the last decade. Different cellulose structures can be used to build up devices that can be used in different medical applications: cellulose nanofibrils, microfibrillated cellulose, microcrystalline cellulose, cellulose nanocrystals and nanowhiskers. Among all of them, cellulose nanocrystals present distinct uses: (1) as drug delivery excipient to connect water-soluble medicine (DOX) or non-ionized hydrophobic anticancer agents (PTX or CUR); (2) as co-stabilizer to improve the physicochemical and flow properties of polymeric excipients [103].

**Figure 16:**
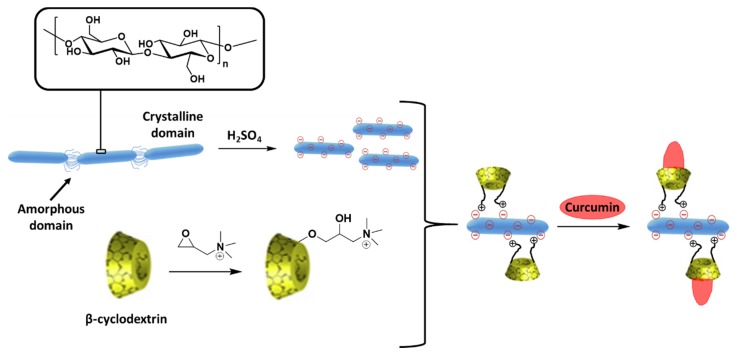
Synthesis pathway for the preparation of cellulose nanocrystals complexes with encapsulated CUR. Adapted with permission from [104]; copyright (2016) Elsevier.

For instance, Ntoutoume et al. described the assembly of CUR-loaded β-cyclodextrin attached to cellulose nanocrystals via electrostatic coupling to obtain ionic complexes. In the resulting compounds, the anticancer drug is inserted within β-cyclodextrin hydrophobic cavities until CUR is released into the tumor (Figure 16). Firstly, the aqueous solubility of CUR increases with the ionic coordination between β-cyclodextrin (which acts as a solubilizing agent) and cellulose nanocrystals. These complexes exhibit tumor targeting properties that enhance the CUR transport into tumor compared with CUR alone when applied to HT29 cells. Furthermore, the presence of the cellulose nanocrystals improved the cell uptake resulting in an increase of the anti-proliferate efficacy [104].

Additionally, the use of different chitosan biobased NCs is described by different researchers for the encapsulation of two different anticancer drugs: Tamoxifen citrate, which is used as drug in breast cancer; and 5-fluorouracil (5-FU), which is used in lung, pancreatic, colon and rectal cancer [105,106]. Moreover, Kathle et al. combined chitosan with gellan gum for the preparation of fully natural-derived nanoparticles [105]. In both cases, the synthesized NCs showed spherical shape and their diameter ranged between tens to hundreds of nanometers. Due to the biodegradability of chitosan, both systems penetrate well in tumor cells than free drug in vitro and they exhibited a better sustained drug release. These improvements make biopolymeric nanoparticles an effective anticancer agent carrier.

Besides, the research on natural products based drugs is increasing in the present because some of the most common anticancer drugs (DOX, PTX, CUR and resveratrol among others) also come from natural resources [107,108].

### 3.9. Overview

Some of the NCs described during this review, the loaded anticancer agent, its applications in cancer therapy and the corresponding reference are summarized in Table 2.

## 4. Conclusions

To sum up, different nanoencapsulated systems involve new scientific routes that are adequate advanced technologies to progress in the detection and diagnostic methodologies of several types of cancers as well as for the delivery of drugs in cancer therapy. Especially, nanoencapsulated systems plays an important role in the delivery of anticancer medicines in pharmaceutical industry.

Since the 1990s, the list of FDA-approved nanotechnology-based products in clinical tests has increased and include: synthetic polymer particles, liposomes, micellar nanoparticles, protein nanoparticles, nanocrystals and many others often in combination with medicines or biologics. 

The use of nanoencapsulation in medicine has contributed to overcome various challenges like toxicity, absorption and/or improved solubility of the used drugs, tumor site targeting, drug resistance, decrease of the required dose and controlled release of the drugs among other advantages. Therefore, the use of nanomedicine revolutionize our ability to diagnose cancer and the cancer therapy. However, the complexity of cellular interactions with the different encapsulated systems and their fate still require more extensive studies to enhance the great impact of nanoencapsulated drug delivery systems on cancer treatment approaches.

## Figures and Tables

**Table 1:** Advantages and drawbacks of nanostructures.

Nanostructure	Advantages	Drawbacks
Inorganic nanoparticles	Facile synthesis Easy surface functionalization Good stability Versatility Exceptional optical and electronic properties	Non-biodegradable Toxicity Coating required
Dendrimers	Synthesis of well-defined structures High chemical and biological stability Efficacy, purity and long shelf life High surface area, loading capacity and targeting Biodegradable and biocompatible	Complex synthetic route Low yield and difficulties in obtaining higher generations
Protein nanoparticles	Low toxicity Biodegradability Good mechanical properties Versatility	Chemical modifications of their surface are usually required to yield stimulus-responsive nanomedicines Low drug loading efficiency
Polymeric micelles	Efficient carrier system for hydrophilic drugs Biodegradable and biocompatible Self-assembling Potential targeting Functional modification Low toxicity	Short circulation times in blood Specific cytotoxicity Need of surface modifications
Liposomes	Amphiphilic structures Easy surface functionalization Biocompatibility	Conventional liposomes: Instability Insufficient drug loading Faster drug release Shorter circulation times in blood
CNTs	Quasi 1D nanostructure Easy surface functionalization Exceptional surface area and cell membrane penetrability Efficient loading Remarkable optical and electronic properties	Poor solubility in many solvents including water Low biodegradability Toxicity
QDs	Good solubility in water after surface modification Strong fluorescence intensity	Non-biodegradable Citotoxicity to lung cells Induction of oxidative stress
Biopolymeric nanoparticles	Isolated from different natural resources (abundance) Excellent geometrical dimensions High specific surface area Good mechanical and barrier properties Lack of toxicity Biodegradable and biocompatible	Hydrophobic materials Poor encapsulation efficiency of medicines Resistance against enzymatic degradation

**Table 2:** Different NCs used in cancer therapy, the loaded anticancer agent and their applications in cancer therapy.

Type of Nanostructure	Anticancer Agent	Applications in Cancer Therapy	Reference
F_3_O_4_ nanoparticles	DOX	Controlled drug delivery system/Magnetic carrier	[37]
Hollow MSNs	DOX	Controlled drug delivery system under the application of an external stimuli (pH)	[41]
(PGLSA)-COONa) dendrimer	10HCPT	Controlled drug delivery system of hydrophobic drugs	[48]
Dendritic phthalocyanine systems	Zinc phthalocyanine	Controlled drug delivery system by photochemical activation	[52]
PAMAM dendrimer coated with Hyaluronic acid	Pt and DOX	Controlled drug delivery system under the application of an external stimuli (pH)	[54]
Gliadin and Gliadin-gelatin nanoparticles	Cyclophosphamide	Controlled release of anticancer drug in breast cancer cells	[57]
Silk nanoparticles	DOX	Controlled drug delivery system under the application of an external stimuli (pH)	[58]
PEG-Polyaspartate micelle	Epirubicin	Controlled drug delivery system under the application of an external stimuli (pH)	[62]
PEG-*b*-PLA micelle	PTX	Controlled drug delivery system of hydrophobic drugs	[63]
Liposome	DOX	Controlled drug delivery system of toxic drugs	[73]
Liposome	CUR	Controlled drug delivery system	[76]
PEG-SWNTs	PTX	Controlled drug delivery system	[85]
SWNTs	Pt	Targeted drug delivery system	[87]
PEG-SWNTs	DOX	Controlled drug delivery system	[88]
Graphene oxide QDs covered with luminescent magnetic nanoplatform	-	Human cancer imaging applications	[92]
Tripeptide-tagged (arginine-glycine-aspartic) CdTe/ZnS QDs	-	Targeted near-infrared imaging of tumors Cancer detection and management including imaging-guided surgery	[95]
ZnO QDs	DOX	Controlled drug delivery system under the application of an external stimuli (pH)	[99]
β-cyclodextrin attached to cellulose nanocrystals	CUR	Targeted and controlled drug delivery system	[104]
Chitosan-gellan gum	Tamoxifen citrate	Controlled drug delivery system	[105]
Chitosan-poly(N-vinylpyrrolidone-alt-itaconic anhydride)	5-FU	Controlled drug delivery system under the application of an external stimuli (pH)	[106]
